# Supplementary material for: De novo design of protease-activatable cytokine prodrugs
Source: bioRxiv. 2026 Sep 22:2026.09.19.752834. Preprint. [Version 1] doi: 10.64898/2026.09.19.752834 (PMC13622076; doi:10.64898/2026.09.19.752834)
Supplement: Supplementary file 1 [file NIHPP2026.09.19.752834v1-supplement-1.pdf]

bioRxiv preprint doi: https://doi.org/10.64898/2026.09.19.752834; this version posted September 22, 2026. The copyright holder for this preprint (which was not certified by peer review) is the author/funder, who has granted bioRxiv a license to display the preprint in perpetuity. It is made available under a CC-BY 4.0 International license.

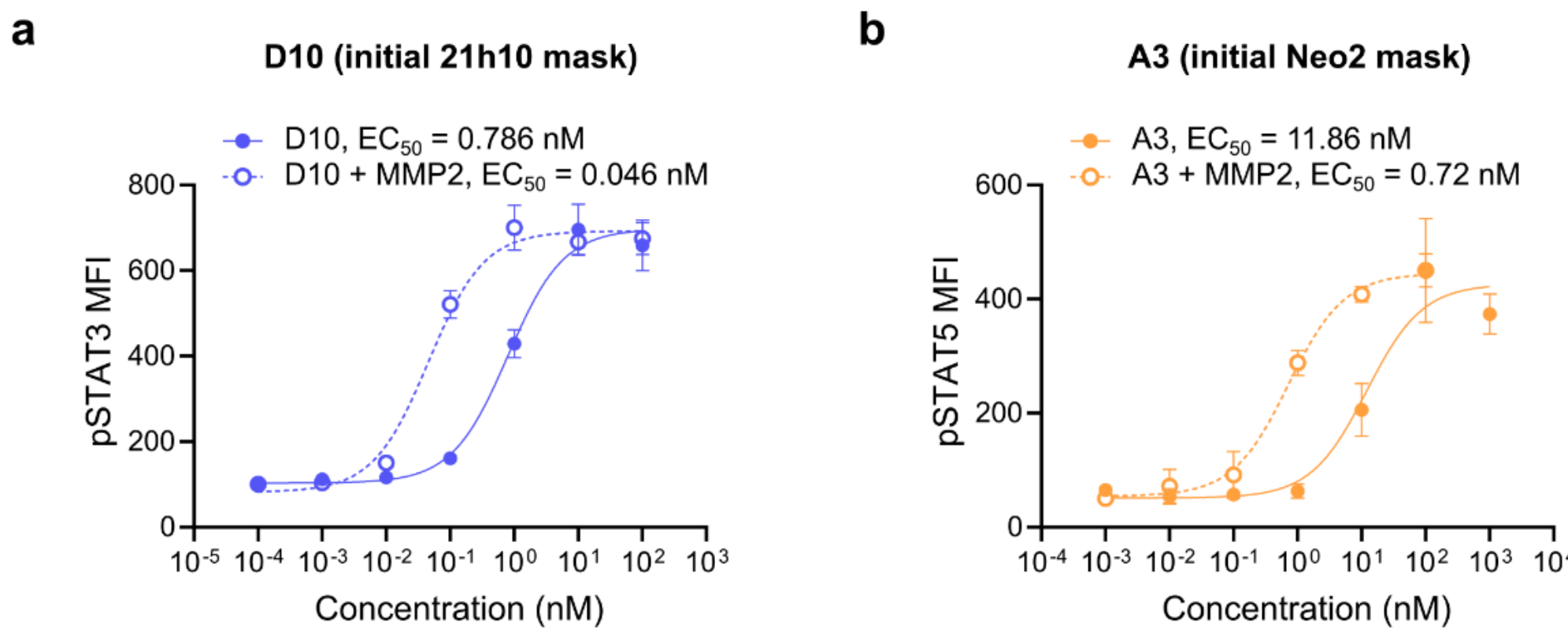

Supplementary Fig. 1. Activity of initial masked IL-21- and IL-2-like cytokine mimics. a,b, Human PBMC signaling assays for D10, the initial 21h10 mask (**a**), and A3, the initial Neo2 mask (**b**), before and after MMP2 treatment. D10 and A3 showed attenuated signaling in the intact masked state and increased activity after proteolytic cleavage. pSTAT3 was measured for D10 signaling, and pSTAT5 was measured for A3 signaling. EC50 values are indicated in each panel.

bioRxiv preprint doi: https://doi.org/10.64898/2026.09.19.752834; this version posted September 22, 2026. The copyright holder for this preprint (which was not certified by peer review) is the author/funder, who has granted bioRxiv a license to display the preprint in perpetuity. It is made available under a CC-BY 4.0 International license.

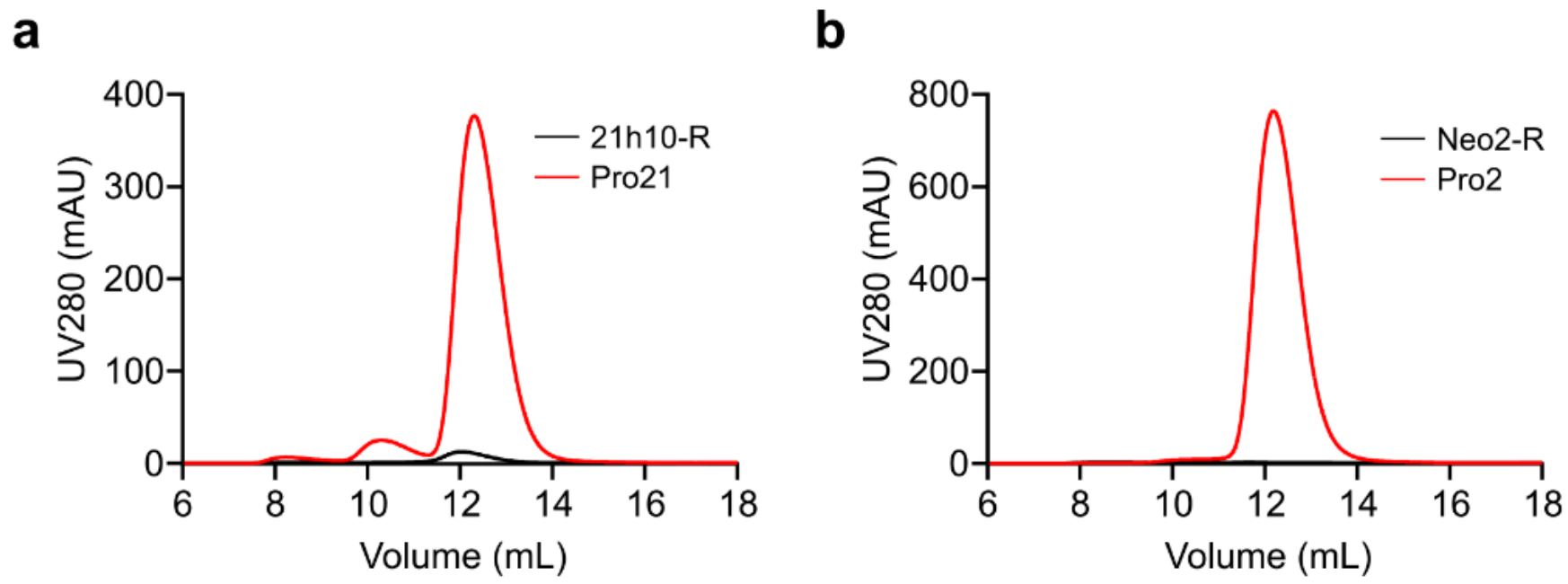

Supplementary Fig. 2. Size-exclusion chromatography analysis of optimized Prokines. **a,b,** Analytical size-exclusion chromatography profiles of purified Pro21 and the corresponding receptor-masked 21h10-R construct (**a**), and Pro2 and the corresponding receptor-masked Neo2-R construct (**b**), monitored by absorbance at 280 nm. Pro21 and Pro2, which contain de novo-designed masking domains, each showed a predominant elution peak, whereas the corresponding receptor-masked constructs showed substantially lower A280 signals under the same production and analysis conditions.

bioRxiv preprint doi: https://doi.org/10.64898/2026.09.19.752834; this version posted September 22, 2026. The copyright holder for this preprint (which was not certified by peer review) is the author/funder, who has granted bioRxiv a license to display the preprint in perpetuity. It is made available under a CC-BY 4.0 International license.

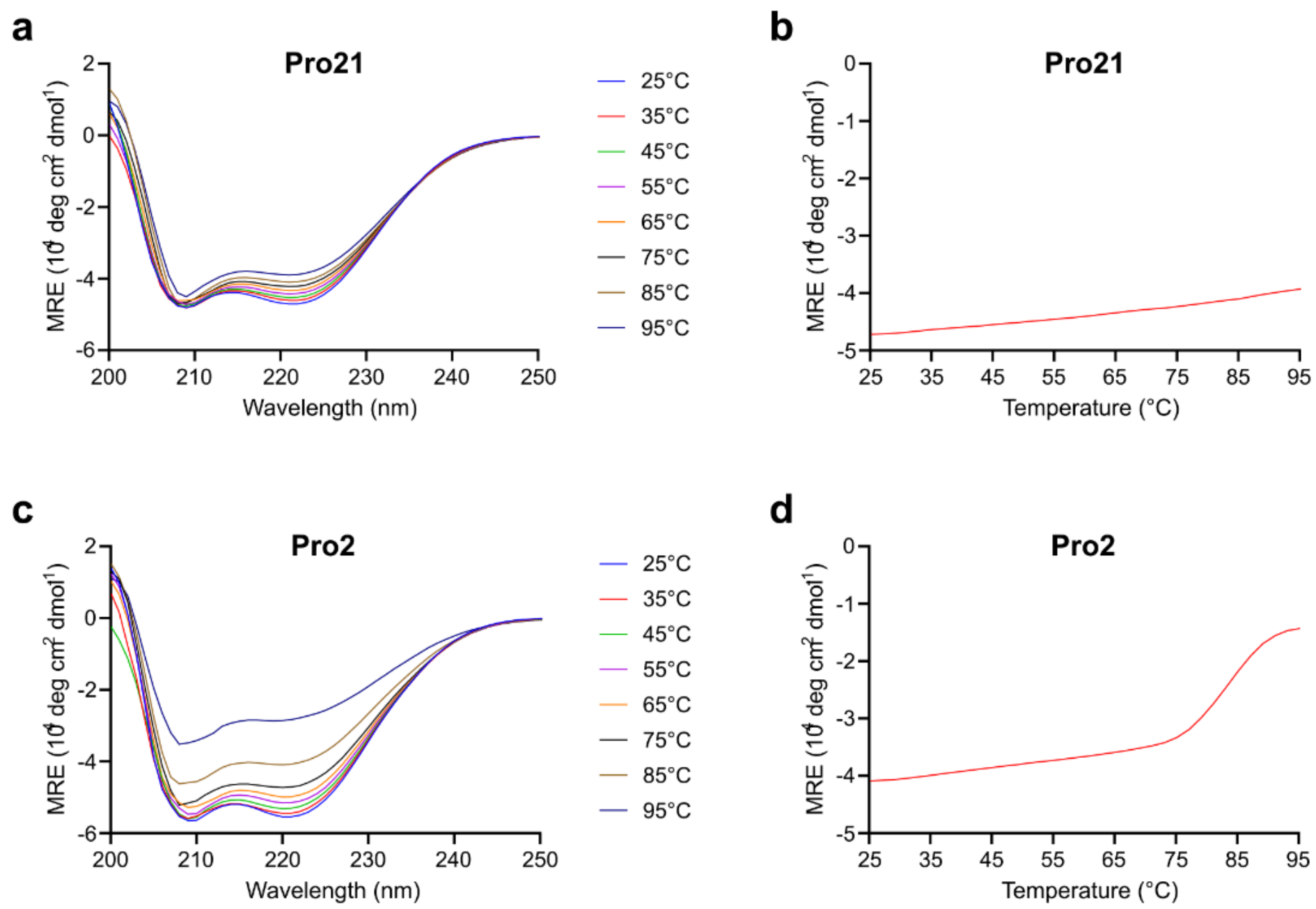

Supplementary Fig. 3. Circular dichroism analysis of Pro21 and Pro2 thermal stability. **a,c,** Far-UV circular dichroism spectra of Pro21 (**a**) and Pro2 (**c**) collected over a temperature range from 25 °C to 95 °C. **b,d,** Temperature-dependent changes in mean residue ellipticity at 222 nm for Pro21 (**b**) and Pro2 (**d**), monitored during thermal ramping.

bioRxiv preprint doi: https://doi.org/10.64898/2026.09.19.752834; this version posted September 22, 2026. The copyright holder for this preprint (which was not certified by peer review) is the author/funder, who has granted bioRxiv a license to display the preprint in perpetuity. It is made available under a CC-BY 4.0 International license.

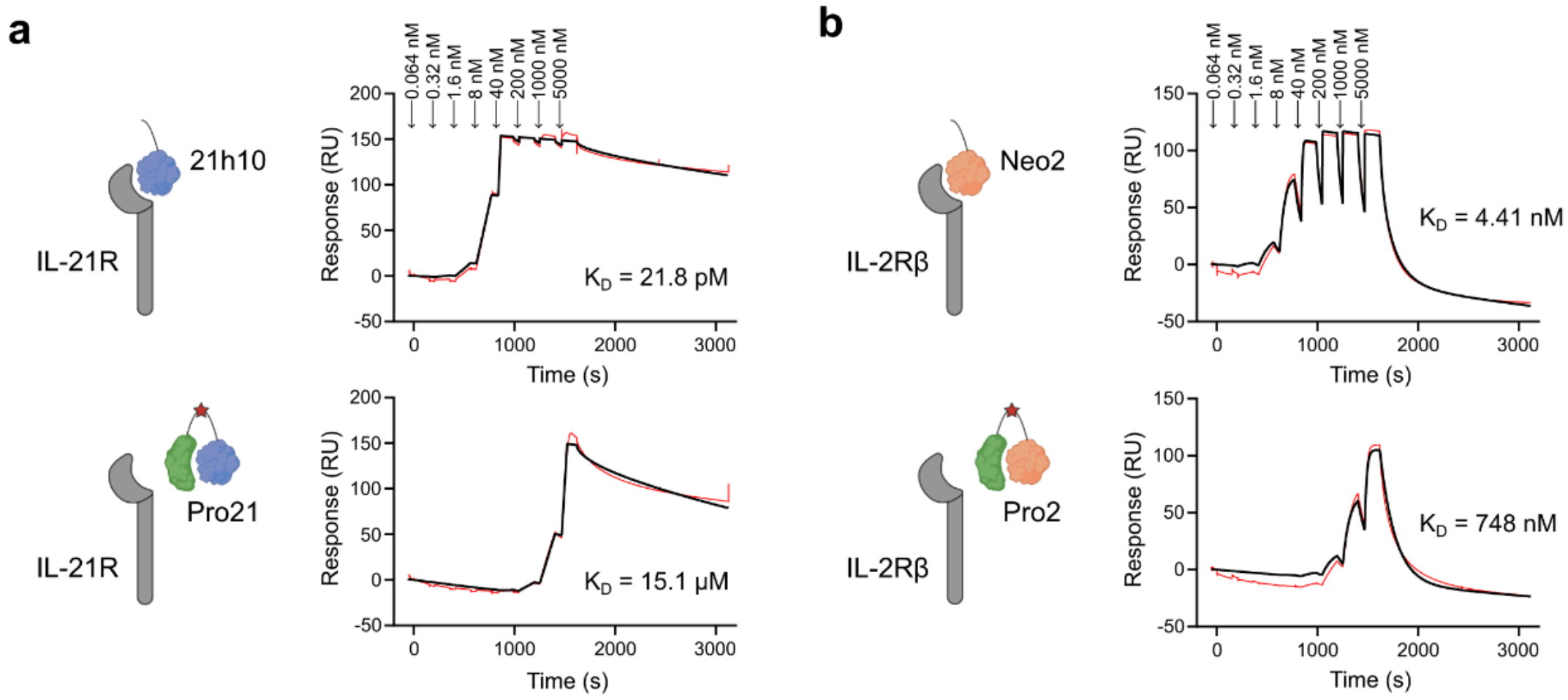

Supplementary Fig. 4. De novo masking reduces receptor binding of Pro21 and Pro2. a, Surface plasmon resonance analysis of 21h10 and Pro21 binding to immobilized IL-21R. Masking reduced IL-21R binding affinity from 21.8 pM for 21h10 to 15.1 μM for Pro21. **b,** Surface plasmon resonance analysis of Neo2 and Pro2 binding to immobilized IL-2Rβ. Masking reduced IL-2Rβ binding affinity from 4.41 nM for Neo2 to 748 nM for Pro2. Black traces indicate experimental sensorgrams and red traces indicate fitted binding curves. K_D values are indicated in each panel.

bioRxiv preprint doi: https://doi.org/10.64898/2026.09.19.752834; this version posted September 22, 2026. The copyright holder for this preprint (which was not certified by peer review) is the author/funder, who has granted bioRxiv a license to display the preprint in perpetuity. It is made available under a CC-BY 4.0 International license.

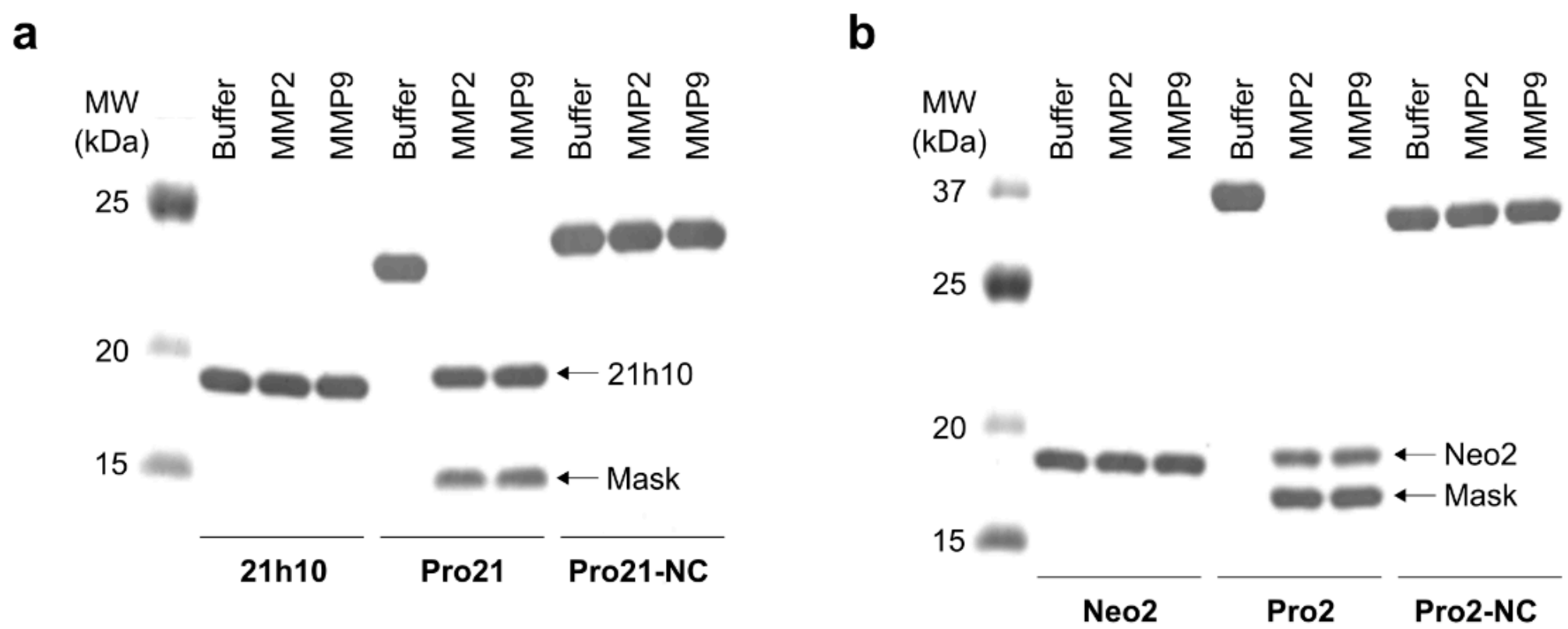

Supplementary Fig. 5. Protease-dependent cleavage of Pro21 and Pro2. a, SDS–PAGE analysis of 21h10, Pro21, and Pro21-NC after incubation with buffer, MMP2, or MMP9. MMP2 and MMP9 treatment cleaved Pro21 to generate bands corresponding to 21h10 and the masking domain, whereas Pro21-NC remained intact. **b,** SDS–PAGE analysis of Neo2, Pro2-M, and Pro2-NC after incubation with buffer, MMP2, or MMP9. MMP2 and MMP9 treatments both cleaved Pro2-M to generate bands corresponding to Neo2 and the masking domain, whereas Pro2-NC remained intact.

bioRxiv preprint doi: https://doi.org/10.64898/2026.09.19.752834; this version posted September 22, 2026. The copyright holder for this preprint (which was not certified by peer review) is the author/funder, who has granted bioRxiv a license to display the preprint in perpetuity. It is made available under a CC-BY 4.0 International license.

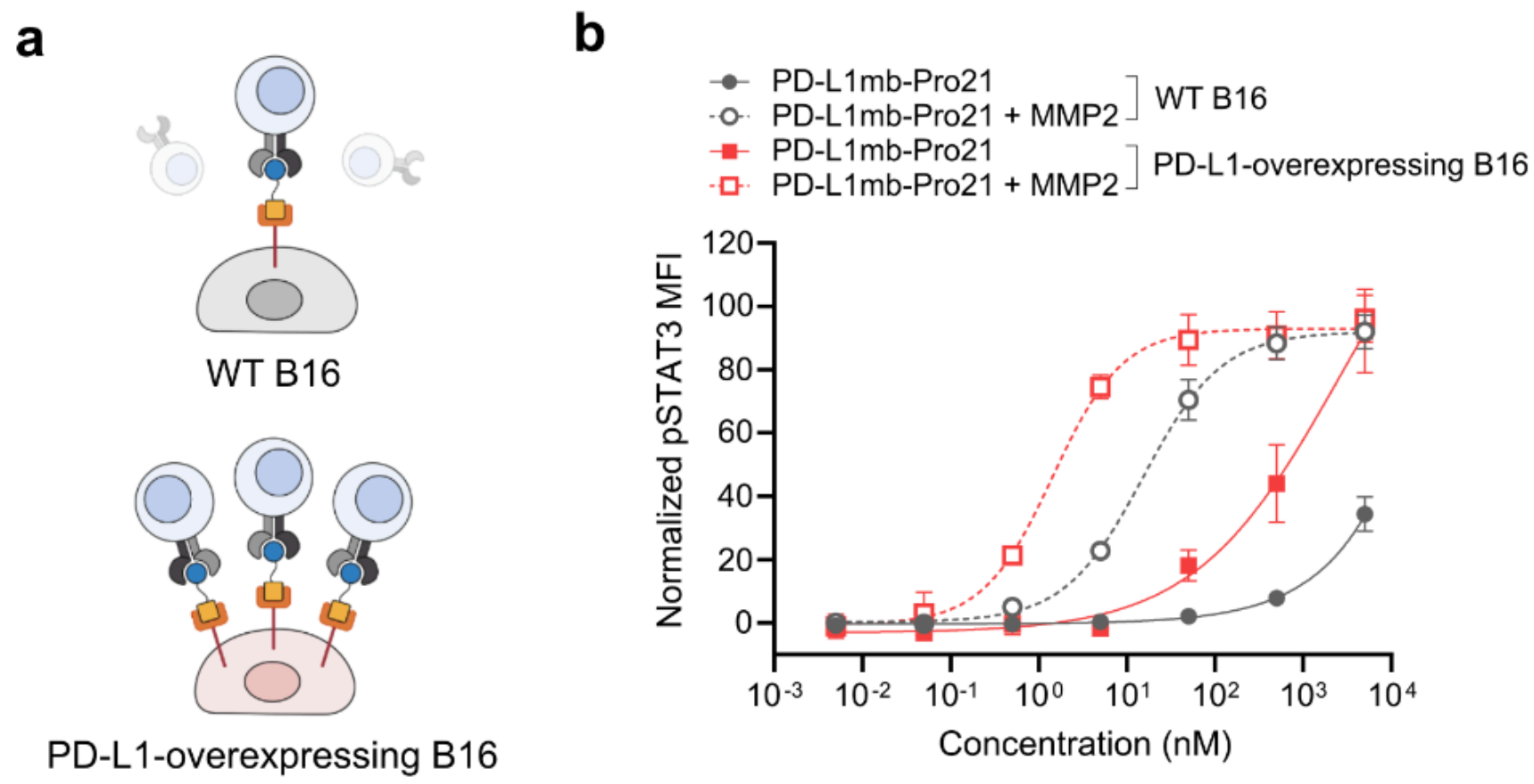

Supplementary Fig. 6. PD-L1 targeting enhances Pro21 activation on PD-L1-expressing cells. a, Schematic of co-culture assay comparing PD-L1-targeted Pro21 activity in the presence of wild-type B16 or PD-L1-overexpressing B16 cells. **b,** Human PBMC signaling assay measuring pSTAT3 activation after treatment with PD-L1mb-Pro21 with or without MMP2. PD-L1-overexpressing B16 cells shifted the response of MMP2-treated PD-L1mb-Pro21 to lower concentrations relative to wild-type B16 cells, consistent with PD-L1-dependent enrichment at the target-cell surface. Data are shown as normalized pSTAT3 mean fluorescence intensity (MFI).

bioRxiv preprint doi: https://doi.org/10.64898/2026.09.19.752834; this version posted September 22, 2026. The copyright holder for this preprint (which was not certified by peer review) is the author/funder, who has granted bioRxiv a license to display the preprint in perpetuity. It is made available under a CC-BY 4.0 International license.

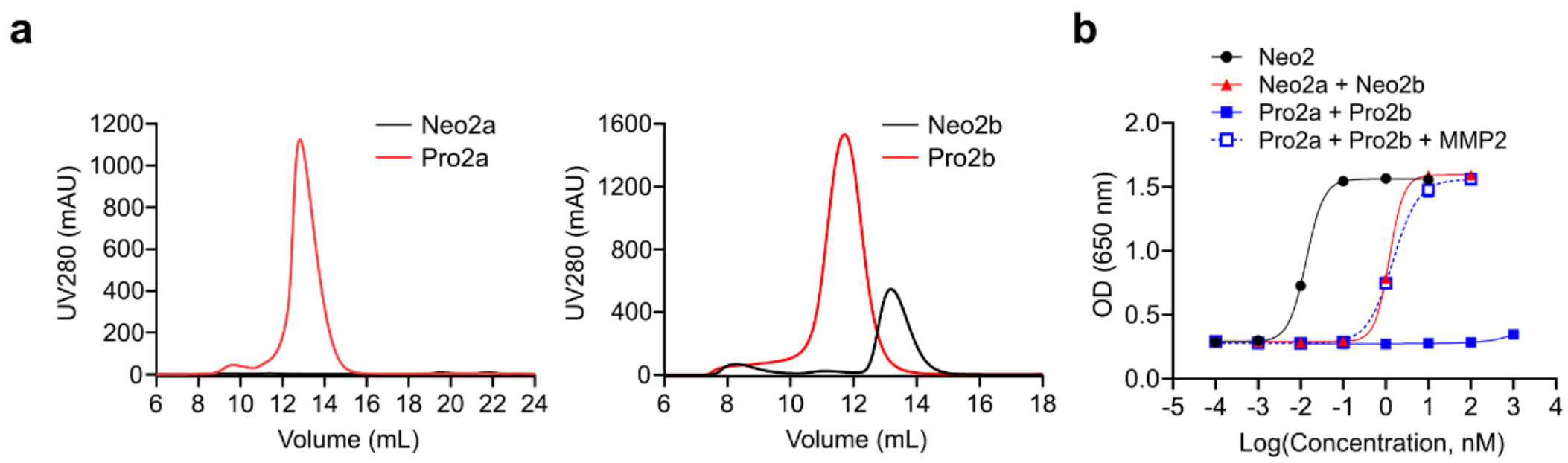

Supplementary Fig. 7. Characterization and protease-dependent activation of masked split Neo2 fragments. a, Analytical size-exclusion chromatography profiles of split Neo2 fragments and corresponding masked fragments. Pro2a and Pro2b eluted as predominant soluble species. **b,** HEK-Blue IL-2 reporter assay comparing full-length Neo2, unmasked split Neo2a plus Neo2b, masked Pro2a plus Pro2b, and MMP2-treated Pro2a plus Pro2b. Masked Pro2a and Pro2b showed minimal activity in the intact state, whereas MMP2 treatment restored activity to a level comparable to the unmasked split Neo2 pair.

bioRxiv preprint doi: https://doi.org/10.64898/2026.09.19.752834; this version posted September 22, 2026. The copyright holder for this preprint (which was not certified by peer review) is the author/funder, who has granted bioRxiv a license to display the preprint in perpetuity. It is made available under a CC-BY 4.0 International license.

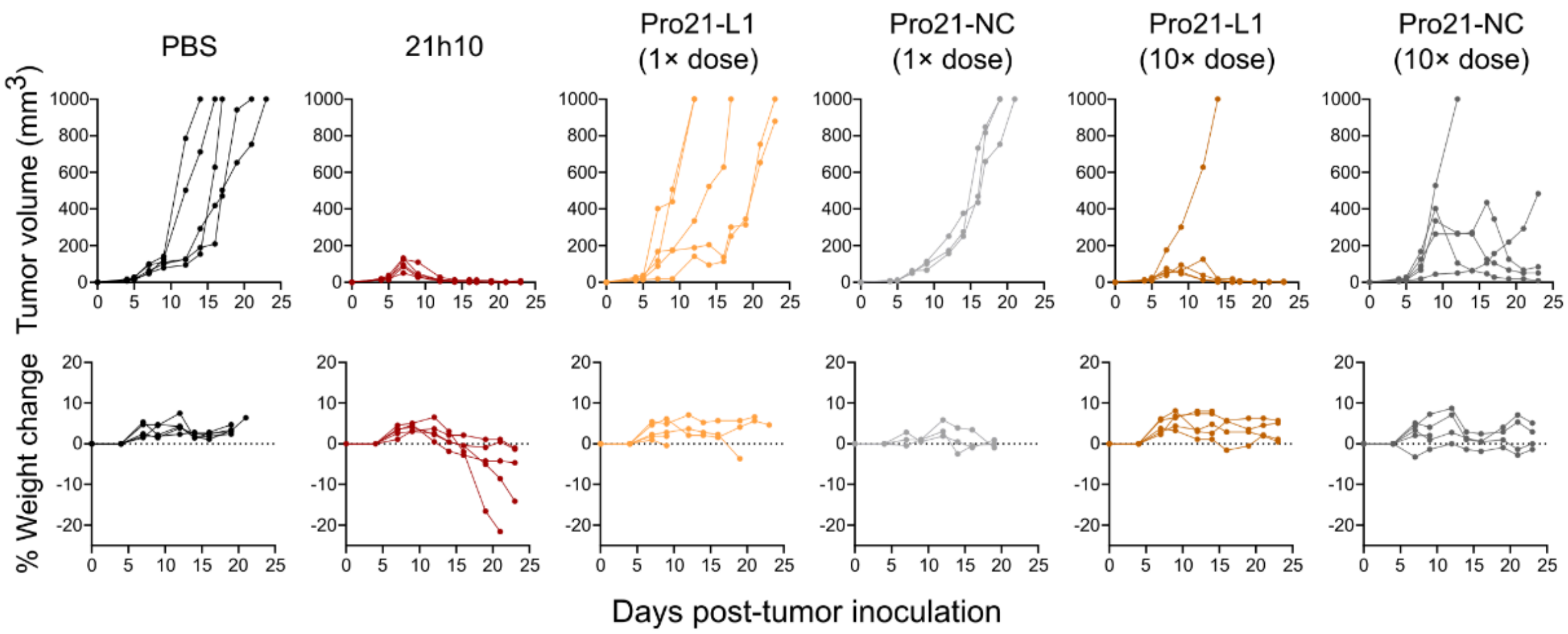

Supplementary Fig. 8. In vivo evaluation of the initial Pro21-L1 construct in the MC38 tumor model. MC38 colon carcinoma-bearing mice were treated with PBS, 21h10, Pro21-L1, or Pro21-NC at 1× or 10× molar-equivalent doses relative to 21h10. Individual tumor growth curves are shown in the top row, and the corresponding percent body-weight changes are shown in the bottom row. 21h10 produced strong tumor control but was associated with body-weight loss. Pro21-L1 reduced body-weight loss relative to 21h10 but showed limited tumor control at the 1× dose, whereas the 10× dose provided greater antitumor activity. The dose-matched Pro21-NC control also showed partial tumor suppression at the 10× dose, suggesting limited cleavage-dependent separation for the initial Pro21-L1 construct under these conditions. n = 5 mice per group.

bioRxiv preprint doi: https://doi.org/10.64898/2026.09.19.752834; this version posted September 22, 2026. The copyright holder for this preprint (which was not certified by peer review) is the author/funder, who has granted bioRxiv a license to display the preprint in perpetuity. It is made available under a CC-BY 4.0 International license.

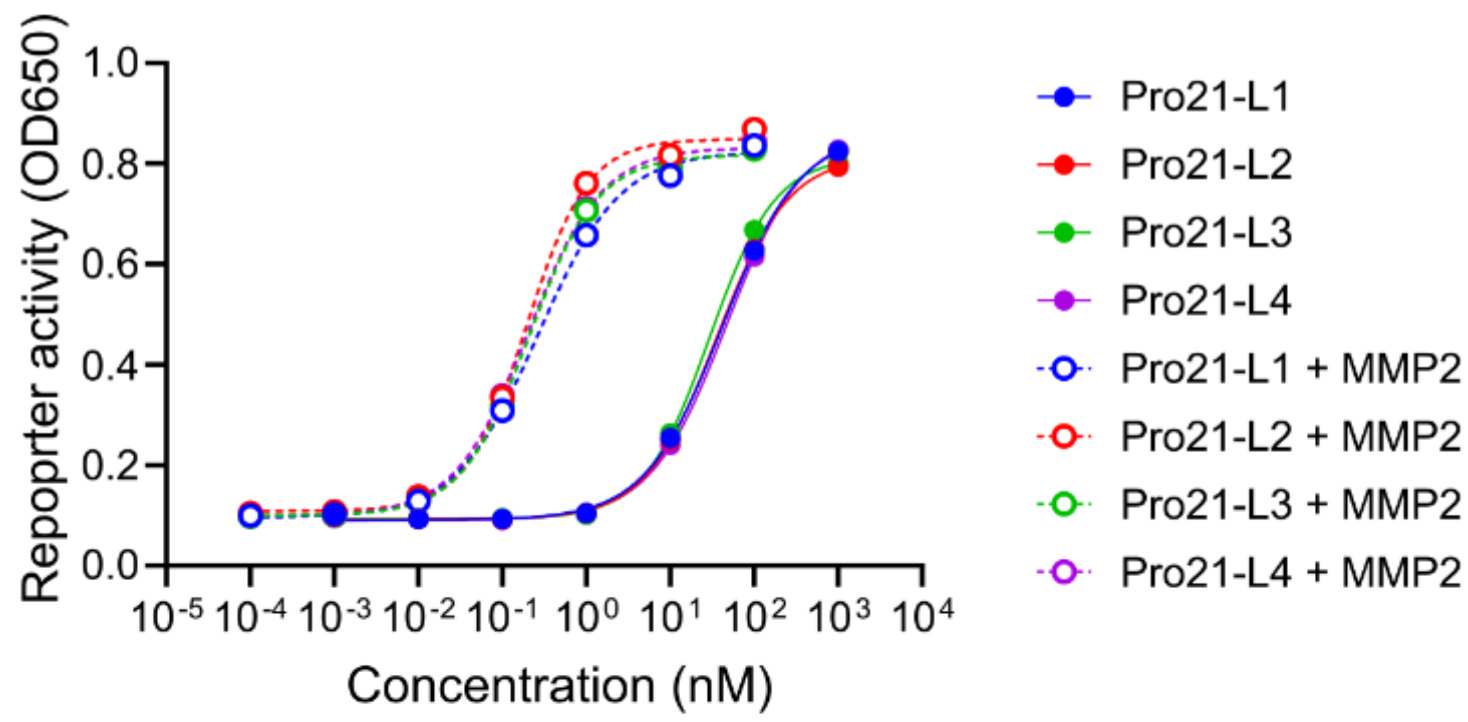

Supplementary Fig. 9. Linker exchange preserves Pro21 masking and protease-dependent recovery. Reporter-cell dose-response assay comparing Pro21-L1, Pro21-L2, Pro21-L3, and Pro21-L4 before and after MMP2 treatment. All linker variants showed similar attenuation in the intact state and comparable recovery of signaling activity following MMP2 treatment, indicating that linker exchange did not substantially alter Pro21 masking or protease-dependent restoration of activity.

bioRxiv preprint doi: https://doi.org/10.64898/2026.09.19.752834; this version posted September 22, 2026. The copyright holder for this preprint (which was not certified by peer review) is the author/funder, who has granted bioRxiv a license to display the preprint in perpetuity. It is made available under a CC-BY 4.0 International license.

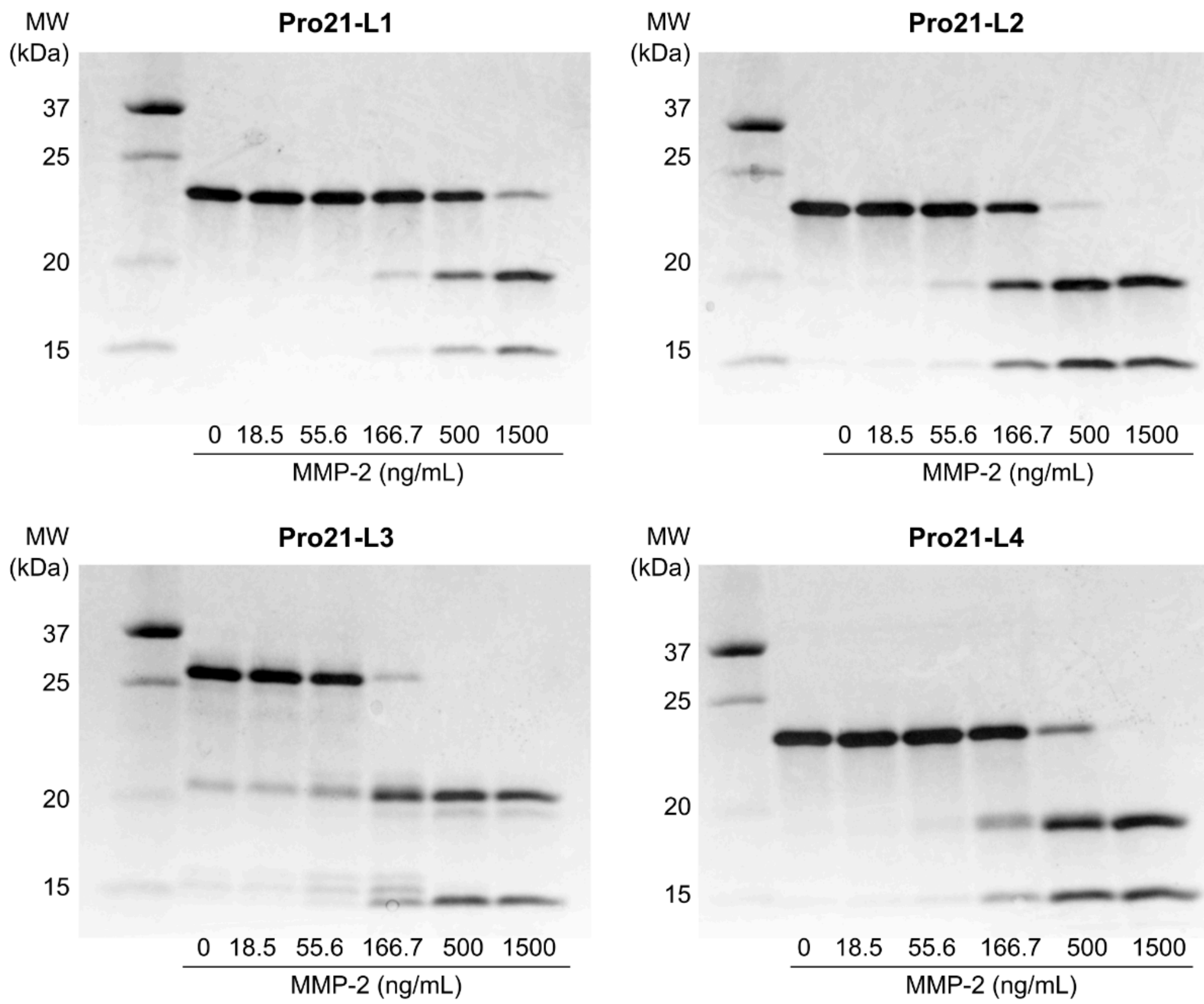

Supplementary Fig. 10. MMP2 sensitivity of Pro21 linker variants. SDS–PAGE analysis of Pro21 linker variants after incubation with increasing concentrations of MMP2. Pro21-L1, Pro21-L2, Pro21-L3, and Pro21-L4 were treated with the indicated concentrations of MMP2, and cleavage was assessed by SDS–PAGE analysis.

bioRxiv preprint doi: https://doi.org/10.64898/2026.09.19.752834; this version posted September 22, 2026. The copyright holder for this preprint (which was not certified by peer review) is the author/funder, who has granted bioRxiv a license to display the preprint in perpetuity. It is made available under a CC-BY 4.0 International license.

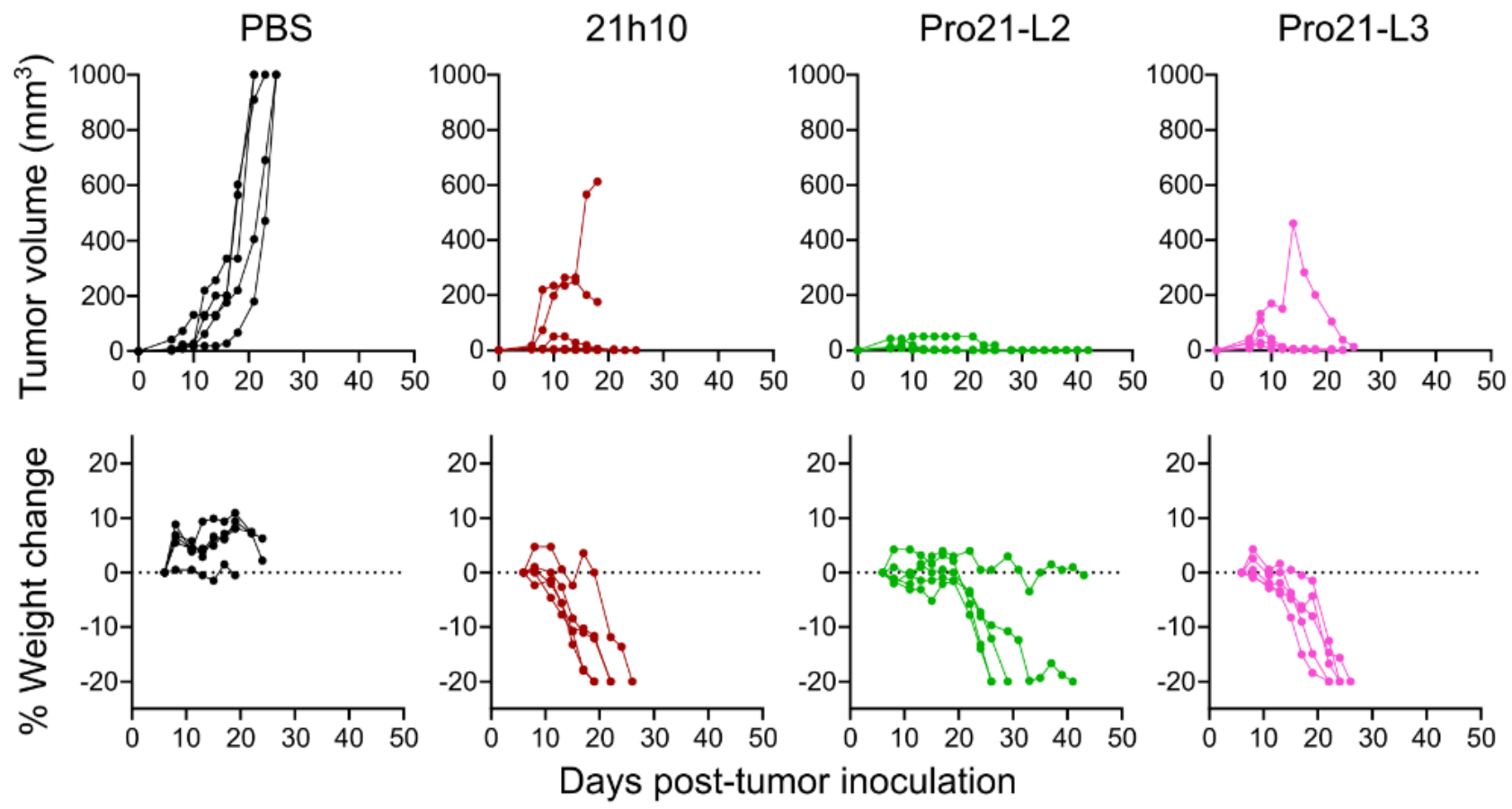

Supplementary Fig. 11. In vivo evaluation of Pro21-L2 and Pro21-L3. MC38 colon carcinoma-bearing mice were treated with PBS, 21h10, Pro21-L2, or Pro21-L3. Individual tumor growth curves are shown in the top row, and the corresponding percent body-weight changes are shown in the bottom row. Pro21-L2 and Pro21-L3 showed strong tumor suppression but were associated with body-weight loss, indicating that increasing linker sensitivity can improve antitumor activity while narrowing the tolerability window. n = 5 mice per group.

bioRxiv preprint doi: https://doi.org/10.64898/2026.09.19.752834; this version posted September 22, 2026. The copyright holder for this preprint (which was not certified by peer review) is the author/funder, who has granted bioRxiv a license to display the preprint in perpetuity. It is made available under a CC-BY 4.0 International license.

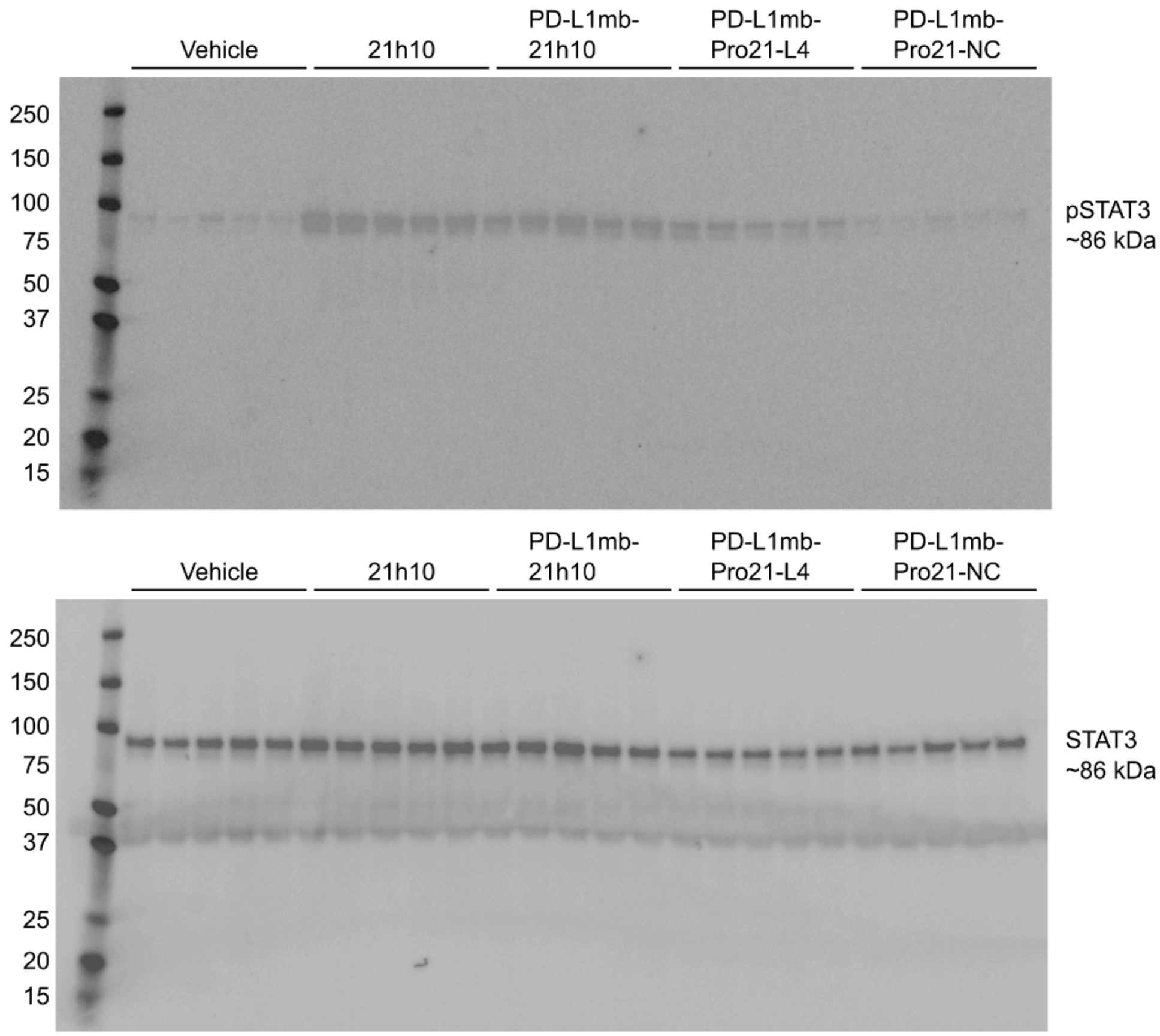

Supplementary Fig. 12. Full immunoblot images for splenic STAT3 signaling. Full immunoblot images corresponding to Fig. 4d showing pSTAT3 and total STAT3 in spleen lysates from the indicated treatment groups. Each lane represents an individual mouse. Molecular-weight markers are indicated in kDa.

bioRxiv preprint doi: https://doi.org/10.64898/2026.09.19.752834; this version posted September 22, 2026. The copyright holder for this preprint (which was not certified by peer review) is the author/funder, who has granted bioRxiv a license to display the preprint in perpetuity. It is made available under a CC-BY 4.0 International license.

Linker ID	Linker sequence	Expected cleavage site	Source
L1	$(GGGS)_2$-(GPLGIAGQ)-$(GGGS)_2$	GPLG↓IAGQ	[33]
L2	$(GGGS)_2$-(HPVGLLAR)-$(GGGS)_2$	HPVG↓LLAR	[12,34]
L3	(GGGS)-$(HPVGLLAR)_3$-(GGGS)	HPVG↓LLAR	[12,34]
L4	(GGGS)-$(GPLGIAGQ)_2$-(GGGS)	GPLG↓IAGQ	[33]
NC	$(GGGS)_6$		

Supplementary Table 1. Protease-cleavable linker sequences used in Prokine constructs. Sequences are shown from N to C in the orientation used between the cytokine mimic and masking domain. Arrows indicate the expected scissile bonds based on previously reported substrate sequences or motif orientation.
